# Growth dynamics of Indian infants using latent trajectory models in pooled survey datasets

**DOI:** 10.3389/fpubh.2024.1474222

**Published:** 2025-01-07

**Authors:** Aswathi Saji, Jeswin Baby, Prem Antony, Srishti Sinha, Sulagna Bandyopadhyay, Joby K. Jose, Anura V. Kurpad, Tinku Thomas

**Affiliations:** ^1^Division of Epidemiology and Biostatistics, St. John’s Research Institute, Bangalore, India; ^2^Department of Statistical Sciences, Kannur University, Kannur, Kerala, India; ^3^Division of Nutrition, St. John’s Research Institute, Bangalore, India; ^4^Department of Physiology, St. John’s Medical College, Bangalore, India; ^5^Department of Biostatistics, St. John’s Medical College, Bangalore, India

**Keywords:** growth trajectory, length-for-age Z score, weight-for-age Z score, longitudinal data, infants

## Abstract

**Background:**

National survey data show that age- and sex-standardized weight and length measurements decline early in Indian children. In population-level longitudinal data, early detection of growth trajectories is important for the implementation of interventions. We aimed to identify and characterize distinct growth trajectories of Indian children from birth to 12 months of age residing in urban and rural areas.

**Methods:**

Pooled data from four interventional and non-interventional longitudinal studies across India were used for the analysis. Latent class mixed modeling (LCMM) was employed to identify groups of children with similar trajectories over age. The trajectories named Classes of Children were created for length-for-age Z scores (LAZ) and weight-for-age Z scores (WAZ) based on place of birth, residential area, and maternal education.

**Results:**

We identified two latent classes for LAZ in boys and three latent classes for LAZ in girls, and four classes for WAZ were identified in both boys and girls. The first class for LAZ, with the highest proportion of children (>80% of children), did not decline or increase with age; In boys, Class 1 was close to the WHO median, whereas in girls, Class 1 was lower than the WHO median from birth. The LAZ classes of remaining boys and girls declined with age (slope, $\mu_{\mathrm{dg}}$ = − 1.04; 95% CI: −1.09, −0.99 for boys and $\mu_{\mathrm{dg}}$ = − 0.69; 95% CI: −0.76, −0.63 for girls). The first trajectory of WAZ (approximately 50% of children) for boys ( $\mu_{\mathrm{dg}}$ =0.13; 95% CI: 0.11, 0.16) and the second trajectory of WAZ for girls ( $\mu_{\mathrm{dg}}$ =0.24; 95% CI: 0.18, 0.30) increased with age, while the remaining trajectories of WAZ declined with age.

**Conclusion:**

There is heterogeneity in the growth of Indian children in the first year of life, which was identified by distinct types of growth trajectories. The predominant trajectories of both LAZ and WAZ did not decline with age, while most other trajectories demonstrated an initial decline.

## Introduction

Low- and middle-income countries (LMICs) continue to report a high prevalence of suboptimal growth (1); for example, 38% of under-five children are stunted in India (2). The first 2 years of a child’s life are crucial to determining their future health and metabolic outcomes. Linear growth faltering and undernutrition in the first 2 years have been linked to poor cognitive, sensory-motor, and social–emotional development, which may reduce economic productivity in adulthood (3–5). Even mild cases of growth faltering have been associated with suboptimal development or delay in developmental milestones (6). Furthermore, there is emerging evidence that growth faltering in early life could also increase cardiovascular risk factors such as glucose intolerance, dyslipidemia, and hypertension in adulthood (7, 8).

Growth in early life can be influenced by multiple factors, including institutional birth, exclusive breastfeeding for the first 6 months, appropriate infant and young child feeding (IYCF) practices, exposure to adequate hygiene and sanitation, maternal education, and morbidities (1, 9, 10). In clinical settings, the growth of children is observed longitudinally, allowing for the assessment of their development over time. However, in public health research, growth has been studied using single or limited anthropometric measurements that can obscure the heterogeneity within population and the ability of children to grow differently, indicating the need for assessing distinct growth trajectories (11). A comparison of weight and height trajectories between well-nourished Pakistani and British children showed differences in these trajectories (12). Indeed, existing evidence suggests that growth trajectories can predict future health-related risks better than a single-point anthropometry-based assessment (13–15).

Cross-sectional growth surveys of Indian children have revealed a significant 0.3 SD decline in World Health Organization (WHO) standard-based length-for-age Z scores (LAZ) from birth to 12 months of age (16), accompanied by a notable decrease in weight-for-age Z-scores (WAZ) from −1.2 SD at birth with a decline of 0.14 SD at 12 months of life. On average, these patterns result in a greater reduction in growth standards at 24 months (mean LAZ at 12 months was −1.07 SD and − 1.32 SD at 24 months, mean WAZ at 12 months was −1.3 SD and − 1.4 SD at 24 months) (16). However, methods examining the association between growth and diet and environmental factors assume that all children are drawn from a single underlying population defined by a set of parameters. Therefore, the present analysis was aimed at comparing the growth velocity of Indian children with the WHO growth velocity standards and characterizing the growth trajectories of Indian children from birth to 12 months of age residing in urban slums and rural areas through multiple longitudinal studies conducted between 1995 and 2006. Additionally, this study focused on identifying potential heterogeneous yet hidden growth patterns. The association between model-based growth trajectory membership and stunting and underweight status at 2 years of age was examined in a subgroup of children. This study aims to inform public health researchers about the use of advanced statistical modeling to identify underlying trajectories such that customized interventions can be considered.

## Methods

### Data sources

The data for the study were accessed through the Healthy Birth, Growth, and Development Knowledge Integration-India (HBGDki) (17), a platform funded by the Bill & Melinda Gates Foundation. This platform enables the researchers to easily access datasets related to Indian children and cohorts. The platform contains 29 survey datasets conducted between 1995 and 2018, focusing on childbirth, growth, and development, including both cross-sectional and longitudinal studies. To model the longitudinal growth of children, we selected four studies from these datasets based on the availability of longitudinal data on anthropometric measures of children, along with related information such as place of residence, mother’s education, and type of delivery.

Longitudinal data at more than two time points from birth to 12 months of age were obtained from the following studies. The data from CMC-V-BC-2002 (Christian Medical College Vellore birth cohort study) (18, 19), SAS Compfeed (SAS Complementary Feeding Practices) (20), SAS Food Supply (SAS-Food supplementation) (21), and ZN-SGA (zinc supplementation in infants born small for gestational age) (22) were combined to form a single dataset; details of individual studies are provided in Table 1. The figure indicating the sample size selection is also in Supplementary Figure 1.

**Table 1:** Description of studies included in the analysis.

Study	Year	Location	Area type	No of children	Intervention	Follow-up period
CMC-V-BC-2002	2002–06	Vellore	Urban slums	373	None	Birth to 54 months (monthly)
SAS Compfeed	1998–99	Haryana	Rural	1,535	Complementary feeding education (2 groups, feeding education vs. control)	Birth to 20 months (once in 3 months)
SAS Food supply	1995–1998	South Delhi	Urban slums	418	Food supplementation (2 groups, Milk-based cereal and nutritional counseling vs. control group)	4 months to 18 months (once in 3 months)
ZN-SGA	1996–98	New Delhi	Urban slums	1997	Zinc supplementation (four groups: zinc, iron, folate, and calcium/phosphorus piboflavin supplementation)	Birth to 21 months (monthly)

The final analytical sample consisted of 2,540 participants for LAZ analysis and 2,690 participants for WAZ analysis, after removing outliers in accordance with WHO standards (23). Outliers were defined as children with LAZ or WAZ beyond ± 6SD at any point in time, as unreliable data at any time point could influence growth trajectories. We opted to examine the growth progression of infants during their first 12 months only, as information was sparse after 12 months of age (17).

### Measurements

#### Anthropometric measurements

Anthropometric measurements of participants from birth to 12 months were considered for growth trajectory analyses. The LAZ and WAZ for each child in the sample were obtained using the WHO growth standards (23). The weight of children was measured using the SECA scale in all studies (accuracy: 0.1 kg), and length was measured using a standardized infantometer (accuracy: 0.1 cm).

#### Early life characteristics

We identified early life characteristics, including institutional birth, maternal education level, residential area, and sex of the child. Residence locations were classified as urban slums and rural areas. Low birth weight was classified as birthweight<2,500 grams. Institutional births were defined as the births that occurred in health facilities. We classified maternal education level based on years of formal education the mother received, where education of more than 12 years was considered ‘college-level,’ less than 12 years was considered ‘school-level,’ and zero years of education was considered ‘not educated.’

### Statistical models

#### Comparison of growth velocity with WHO standards

The longitudinal growth data were compared with WHO growth trajectory standards to understand how the current data were positioned in the trajectory centiles. 1-month weight increments and 2-month length increments were obtained using smoothed monthly anthropometry data. These were compared with the WHO growth velocity standards (23). We calculated the monthly Z-score of weight and length velocities, separately for boys and girls, using the WHO LMS (lambda-mu-sigma) parameters. These parameters are part of the Box-Cox power exponential transformation used in growth standard data construction and we plotted them alongside the WHO standard for visual examination.

#### Growth trajectory analysis

We used latent class mixed models (LCMM) to identify distinct latent trajectories of LAZ or WAZ in longitudinal data from birth to 12 months for boys and girls separately (24). The LCMM assumes that the population of children is heterogeneous in terms of z-scores, and G latent classes are used to represent these groups. Latent growth trajectories that represent a pattern of change in z-scores were identified as follows.

The procedure to define the growth trajectory of infants is as follows. Consider 𝑁 children followed up on multiple and different occasions up to the age of 12 months. Let, $Y_{ij}=\left(Y_{i1},Y_{i2},\ldots ,Y_{in_{i}}\right)$ be the anthropometry Z scores for $n_{i}$ follow-ups of the $i^{th}$ child. Let $X_{ci},c=1,2\ldots C$ be the C covariates (early life characteristics) with fixed effects, and $Z_{dij},d=1,2\ldots D$ be the D independent variables (age in months as linear or non-linear terms) whose effect is assumed to vary for each child and each latent group.

Let $\pi_{ig},g=1,2\ldots G$ be the probability that the child is included in a latent group g . Considering the G^th^ class as the reference class, the probability of i^th^ child belonging to the g^th^ class is described using a multinomial logistic model according to $X_{ci}$ .


$$\pi_{ig}=\frac{\exp\left(\alpha_{0g}+\sum_{c=1}^{C}X_{ci}\alpha_{1g}\right)}{\sum_{g=1}^{G}\exp\left(\alpha_{0g}+\sum_{c=1}^{C}X_{ci}\alpha_{1g}\right)}$$

Based on the maximum class membership probability, each child was classified into either of the G latent growth classes. In an LCMM framework, $Y_{ij}$ is given by:

$Y_{ij}=\overset{G}{\underset{g=1}{\sum }}\beta_{0gi}+\overset{C}{\underset{c=1}{\sum }}\beta_{c}X_{ci}+\overset{D}{\underset{d=1}{\sum }}\overset{G}{\underset{g=1}{\sum }}\mu_{dgi}Z_{dij}+\varepsilon_{ij}$ _,_ $\varepsilon_{ij}\sim N\left(0,\sigma_{\varepsilon }^{2}\right)\text{.}$

$\beta_{0gi}=\beta_{0g}+u_{gi}$ , $u_{gi}\sim N\left(0,\sigma_{u}^{2}\right)$

$\mu_{dgi}=\mu_{\mathrm{dg}}+v_{gi}$ , $v_{gi}\sim N\left(0,\sigma_{v_{d}}^{2}\right)$

Here, $\beta_{0gi}$ is the intercept for the *g^th^* trajectory class and $\mu_{dgi}$ is the slope. $X_{c}$ is the early life characteristics considered for the analysis (place of birth, residential area, and maternal education), and we selected d as 2, assuming that age is linearly and quadratically assumed to vary for each child and each trajectory. We obtained the estimates of model parameters within a maximum likelihood framework using the modified Marquardt algorithm (25). Bayesian Information Criteria (BIC) and Log-likelihood were the criteria used to determine the number of trajectories (26). We also considered a minimum trajectory membership of 5% in the smallest group as another criterion for determining distinct trajectories (27). Visualization of growth trajectories was useful in determining the number of classes, assessing the quality of classification, and understanding the growth pattern of children in different classes (28). All available data were considered in the mixed effects model with individuals as the random effect, and missing data were not imputed.

#### Association of trajectories with anthropometric outcomes

The following analysis was performed to examine how the outcome of latent class trajectories of LAZ and WAZ until 12 months of age affect the poor nutritional status of the children at 2 years. Logistic regression models were used to predict the nutritional status at 2 years with the latent class membership of the children based on the LCMM model. According to the LCMM model, the child’s LAZ trajectory class was considered the predictor in the logistic regression model for stunting, and the WAZ trajectory class to which the child belonged was considered as the predictor in the logistic regression model for underweight status. The child’s birth weight and maternal education were considered covariates in the analysis. A receiver operating characteristic (ROC) analysis of prediction probability from the logistic regression model was used to determine the optimal threshold to classify children as either stunted, underweight, or normal. Sensitivity, specificity, and accuracy (overall % of correct classification) of nutritional status classification were used to assess the model’s predictive performance. K-fold cross-validation was used to validate the logistic model. All analyses were carried out using the packages LCMM v1.9.2 and R v4.0.2 software.

## Results

The characteristics of the children included in this study are summarized in Table 2. In the data considered for LAZ modeling, 50% of the children were girls, 51% were from urban slums, 72% were delivered at home, and 55% of the mothers were ‘not educated.’ The distribution of LAZ and WAZ in the four cohorts is given in Supplementary Figure 2. The average LAZ of the children at birth were − 0.98 ± 1.25 for boys and − 0.93 ± 1.18 for girls, while the average WAZ of the children at birth were − 1.29 ± 1.09 for boys and − 1.42 ± 1.12 for girls.

**Table 2:** Descriptive characteristics of children.

Variable	Category	Sample considered for LAZ (*N* = 2,540)	Sample considered for WAZ (*N* = 2,690)
		N (%)	N (%)
Sex	Girls	1,271 (50.0)	1,354 (50.3)
	Boys	1,269 (50.0)	1,336 (49.7)
Residence type	Rural	1,235 (48.6)	1,242 (46.2)
	Urban slums	1,305 (51.4)	1,448 (53.8)
Place of birth	Home	1836 (72.3)	1944 (72.3)
	Institutional	704 (27.7)	746 (27.7)
Mother’s education level	College	30 (1.2)	34 (1.3)
	Schooling	1,100 (43.3)	1,160 (43.1)
	Illiterate	1,410 (55.5)	1,496 (55.6)

LAZ, Length for Age Z score; WAZ, Weight for Age Z score.

### Comparison of growth velocity with WHO standards

**Figure 1:**
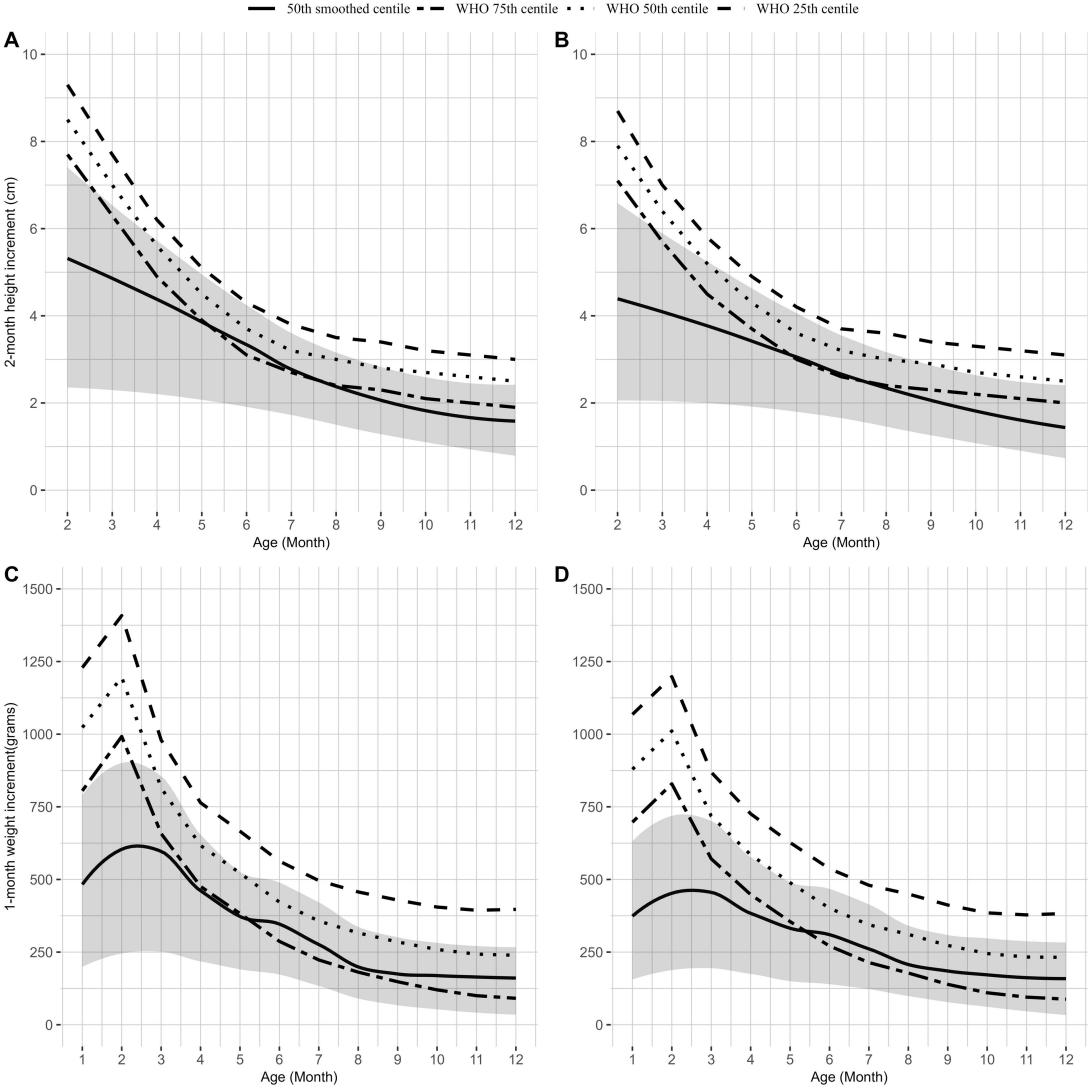
Comparison of WHO length velocity percentiles and percentiles of length velocity for boys **(A)** and girls **(B)**. Comparison of WHO weight velocity percentiles and percentiles of weight velocity for boys **(C)** and girls **(D)**. The dotted line represents the 50^th^ percentile WHO growth velocities of weight and length, and the dot-dashed line represents the 25th percentile and 75th percentile of WHO weight and length velocities. The solid line represents the 50th percentile weight and length velocity from our data with a shaded confidence interval (25th percentile and 75th percentile).

The 2-month length velocity calculated based on WHO standard LMS parameters was found to be low ( < -2SD) until 3 months of age with subsequent improvement (Supplementary Table 1) for both boys and girls. However, this improvement fails to reach the median or even the 25^th^ percentile of standard velocity recommended for infants in this age group as per WHO standards (Figures 1A,B). The monthly average weight velocity improved from 1 month to 12 months of age based on WHO growth velocity standards, with the z-score mostly remaining ≤ -1.0SD up to 4 months of age and eventually improving to −0.35 SD at 12 months of age (Supplementary Table 2) for boys and − 0.3 SD for girls. The smoothed 50^th^ percentile values for one-month weight gain remained below the median WHO growth velocity standards at all ages for both boys and girls. This gain in weight can be observed in Figures 1C,D.

### Growth trajectory analysis

The trajectory model fit statistics for LAZ and WAZ are given in Supplementary Table 3. Latent models with three trajectories fulfilled the criteria of minimum BIC value and the minimum 5% trajectory membership criteria for both LAZ and WAZ in boys and girls.

**Figure 2:**
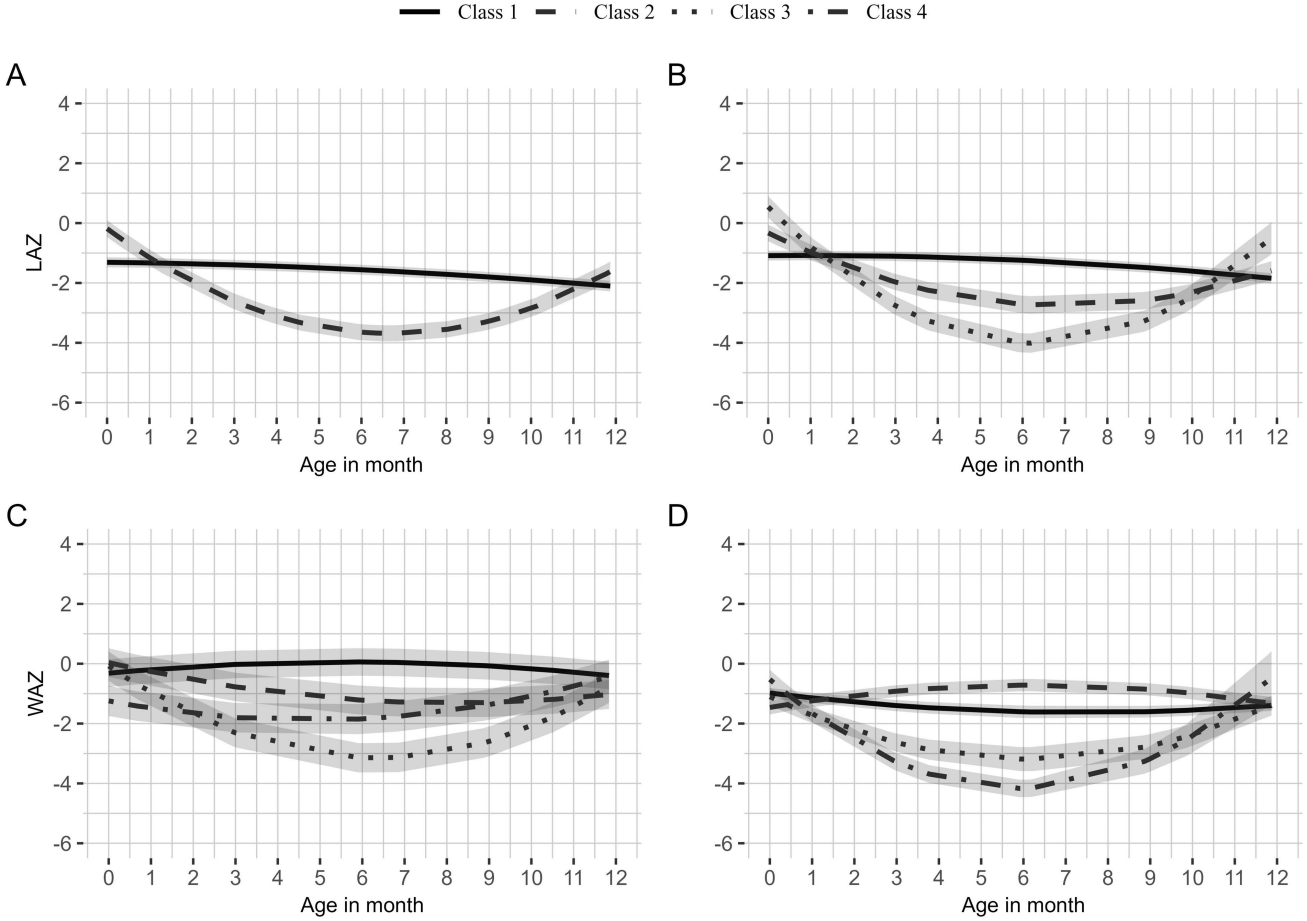
Latent class growth trajectories of length-for-age z-scores (LAZ) for boys **(A)** and girls **(B)** and weight-for-age z-scores (WAZ) for boys **(C)** and girls **(D)**. Solid lines represent the first class with the highest proportion; dashed lines represent the second class with the second-highest proportion; dotted lines represent the third class with the third-highest proportion; and dot-dashed lines represent the last class with the lowest proportion of children.

For boys LAZ, 83.7% belonged to Class 1 (Supplementary Table 3), characterized by LAZ at birth that was close to the WHO median $(\beta_{0g}=$ −0.4 SD, *p* > 0.05) with no decline over time ( $\mu_{\mathrm{dg}}$ = − 0.02; 95% CI: −0.035, 0.003) as they aged (Table 3; Figure 2A). The remaining 16.3% belonged to Class 2, who had higher LAZ at birth ( $\beta_{0g}$ =0.72), but this score declined with age $(\mu_{\mathrm{dg}}$ = − 1.04; 95% CI: −1.09, −0.99).

**Table 3:** Estimates of trajectory models for identifying latent groups of LAZ.

Characteristics		Boys Est (95% CI)	Girls Est (95% CI)
Fixed effects			
Residential area	Rural	*Ref*	
	Urban Slum	−0.21 (−0.36, −0.08)	−0.16 (−0.29, −0.02)
Place of birth	Institutional	*Ref*	
	Home	−0.18 (−0.32, −0.04)	−0.40 (−0.53, −0.27)
Mother’s education level	College	*Ref*	
	Schooling	−0.6 (−1.06, −0.14)	0.06 (−0.51,0.63)
	Illiterate	−0.91 (−1.37, −0.44)	−0.2 (−0.78,0.38)
Mixture effects			
Class-specific intercept	Class 1	−0.40 (−0.87,0.06)	−0.89 (−1.46, −0.32)
	Class 2	0.72 (0.22,1.23)	−0.13 (−0.75,0.49)
	Class 3	–	0.74 (0.09,1.4)
Class-specific slope due to age	Class 1	−0.02 (−0.035,0.003)	0.01 (−0.01,0.033)
	Class 2	−1.04 (−1.09, −0.99)	−0.7 (−0.76, −0.63)
	Class 3	–	−1.44 (−1.53, −1.35)
Class-specific slope due to age^2^	Class 1	−0.004 (−0.006, −0.003)	−0.006 (−0.008, −0.005)
	Class 2	0.078 (0.07,0.08)	0.05 (0.04,0.06)
	Class 3	–	0.11 (0.11,0.12)
Random effects			
intercept	_	1.11 (1.00,1.22)	0.92 (0.83,1.02)
Age	_	0.011 (0.009,0.012)	0.009 (0.007,0.01)
Error	_	0.69 (0.68, 0.7)	0.66 (0.65, 0.67)

The LAZ for girls was categorized into three classes. Class 1 comprised 80.2% and Class 2 comprised 13.3% of the girls (Supplementary Table 3). Class 1 (Table 3; Figure 2B) had a low LAZ at birth ( $\beta_{0g}=$ −0.9 SD) and no decline with age ( $\mu_{\mathrm{dg}}$ =0.01; 95% CI: −0.01, 0.033), while Class 2 had LAZ close to WHO median ( $\beta_{0g}=$ −0.13 SD, *p* > 0.05) at birth and this declined with age ( $\mu_{\mathrm{dg}}$ = − 0.7; 95% CI: −0.76, −0.63) until 6 months, after which there was a slight improvement ( $\mu_{\mathrm{dg}}$ =0.05; 95% CI: 0.04, 0.06). Finally, Class 3 (which constituted 6.5% of the girls) had a high LAZ ( $\beta_{0g}=$ 0.74 SD) at birth and experienced a significant decline with age until 6 months ( $\mu_{\mathrm{dg}}$ = − 1.44; 95% CI: −1.53, −1.35) and catch-up growth by 12 months ( $\mu_{\mathrm{dg}}$ = 0.11; 0.11, 0.12) as observed from the quadratic model.

Four WAZ trajectory classes were identified for boys and girls from birth to 12 months of age (Supplementary Table 3). For boys, the WAZ at birth for Class 1 (46.1%) was close to the WHO median ( $\beta_{0g}=$ −0.32 SD, *p* > 0.05) and slightly increased (Table 4; Figure 2C) with age ( $\mu_{\mathrm{dg}}$ =0.13; 95% CI: 0.11, 0.16). Class 2 also had WAZ close to the WHO median at birth, but this declined significantly with age ( $\mu_{\mathrm{dg}}$ = − 0.33; 95% CI: −0.37, −0.29). Class 3 was similar to class 2 but significantly declined with age ( $\mu_{\mathrm{dg}}$ = − 0.98; 95% CI: −1.02, −0.94). Class 4 comprised only 5.8% of infants with low WAZ at birth $(\beta_{0g}$ = − 1.24; 95% CI: −1.75, −0.74), and it declined with age $(\mu_{\mathrm{dg}}$ = − 0.27; 95% CI: −0.36, −0.19). All classes showed slight improvement in WAZ after 6 months except Class 1.

**Table 4:** Estimates of trajectory models for identifying latent groups of WAZ.

Characteristics		Boys Est (95% CI)	Girls Est (95% CI)
Fixed effects			
Residential area	Rural	*Ref*	
	Urban Slum	−0.17 (−0.32, −0.02)	−0.29 (−0.42, −0.17)
Place of birth	Institutional	*Ref*	
	Home	−0.22 (−0.35, −0.09)	−0.49 (−0.62, −0.37)
Mother’s Education Level	College	*Ref*	
	Schooling	−0.85 (−1.31, −0.39)	−0.13 (−0.64, 0.38)
	Illiterate	−1.09 (−1.55, −0.63)	−0.34 (−0.85, 0.16)
Mixture effects			
Class-specific Intercept	Class 1	−0.32 (−0.78, 0.14)	−0.64 (−1.14, −0.13)
	Class 2	0.04 (−0.44, 0.52)	−1.12 (−1.68, −0.56)
	Class 3	−0.08 (−0.56, 0.41)	−0.76 (−1.34, −0.18)
	Class 4	−1.24 (−1.75, −0.74)	−0.18 (−0.77, 0.41)
Class-specific slope due to Age	Class 1	0.13 (0.11, 0.16)	−0.18 (−0.23, −0.13)
	Class 2	−0.33 (−0.37, −0.29)	0.24 (0.18, 0.30)
	Class 3	−0.98 (−1.02, −0.94)	−0.68 (−0.81, −0.55)
	Class 4	−0.27 (−0.36, −0.19)	−1.23 (−1.34, −1.12)
Class-specific slope due to Age2	Class 1	−0.012 (−0.014, −0.01)	0.012 (0.01, 0.02)
	Class 2	0.021 (0.017, 0.024)	−0.02 (−0.024, −0.015)
	Class 3	0.077 (0.074, 0.081)	0.055 (0.046, 0.064)
	Class 4	0.03 (0.02, 0.04)	0.10 (0.09, 0.12)
Random Effects			
Intercept	_	0.83 (0.75, 0.92)	0.92 (0.83, 1)
Age	_	0.006 (0.005, 0.007)	0.009 (0.008, 0.01)
Error	_	0.55 (0.5405, 0.5601)	0.545 (0.535, 0.554)

The Class 1 trajectory (52.8%) for girls was similar to the Class 1 WAZ trajectory for boys (Table 4; Figure 2D). Although Class 2 had a low WAZ at birth, an increment was observed with age ( $\mu_{\mathrm{dg}}$ =0.24, 95% CI: 0.18, 030). Class 3 (13.5%) had a low WAZ at birth ( $\beta_{0g}=$ −0.76 SD), which declined with age $(\mu_{\mathrm{dg}}$ = − 0.68; 95% CI: −0.81, −0.55). Class 4 (6.9%) of girls had WAZ close to the WHO median at birth, which significantly declined with age $(\mu_{\mathrm{dg}}$ = − 1.23; 95% CI: −1.34, −1.12). All classes demonstrated a slight improvement in WAZ after 6 months except Class 1 and Class 2.

We compared the latent class assignment of the children between the intervention groups of the three included studies separately. It was observed that latent class assignment was not associated with the intervention the children received in the different studies, except for LAZ in girls of the Zinc study (see Supplementary Table 4).

### Predictors of growth trajectories

According to the LCMM analysis, residential location (urban slum vs. rural) and place of birth were significant predictors for identifying both boys’ and girls’ LAZ and WAZ trajectories (Tables 3, 4). Additionally, maternal education was a significant predictor of LAZ and WAZ trajectories only in boys. The covariates contributing to LAZ and WAZ class-specific membership of the infants are given in Supplementary Tables 5, 6. The distribution of covariates by latent classes of LAZ and WAZ are given in Supplementary Tables 7, 8. In general, the classes in which an initial decline in LAZ and WAZ was observed (Class 2 and above) consisted mostly of urban slum children (>90%). Additionally, these classes mostly included children who had ‘not educated’ mothers (>60% for LAZ and nearly 50% for WAZ).

### Association between trajectories and anthropometric outcomes

Length data at 2 years were available for 170 boys and 173 girls, and weight data was available for 180 boys and 185 girls (24th or 25th month). The prevalence of stunting was 73.5% and that of underweight was 43.9% in boys. In girls, the prevalence of stunting and underweight was 64.2 and 38.4%, respectively. The latent classes of LAZ and WAZ were not associated with stunting and underweight at 2 years (Supplementary Table 9). The sensitivity of stunting and underweight classification based on the growth trajectory followed was low (Table 5), at <60%, except for the prediction of underweight in boys. The specificity was >60% for both stunting and underweight in boys and girls.

**Table 5:** Accuracy of classifications in the predictive model.

Model	Prevalence at the end of 2 years (%)	Accuracy (%)	Sensitivity (%)	Specificity (%)
Stunting—boys	73.53	57.65	54.4	66.67
Stunting—girls	64.16	59.54	57.66	62.9
Underweight—boys	43.89	72.22	70.89	73.27
Underweight—girls	38.38	60.54	57.75	62.28

## Discussion

The present analysis showed a lower growth velocity for both length and weight than the normative WHO growth velocity among Indian infants. There were two and three distinct LAZ trajectories for boys and girls, respectively, while there were four WAZ trajectories for both boys and girls. The LAZ of children in the predominant trajectory did not decline with age, while the WAZ of children in the predominant trajectory increased with age. Residential area and institutional delivery were the predictors of LAZ and WAZ trajectories for both boys and girls. Maternal characteristics, such as height and BMI, significantly influenced child growth outcomes and trajectories of LAZ in children from LMICs (29). In this analysis, maternal education was also identified as a critical factor in shaping both LAZ and WAZ trajectories in boys.

The classification of growth using the WHO growth standards indicates growth deprivation at the population level but may overlook children with the potential for improvement. The growth velocity observed in this study was comparable to that reported in secondary analyses of cohorts across LMICs (30). Therefore, we attempted to identify if different trajectories could differentiate children with the potential to grow better in a group of children who were born and brought up in suboptimal conditions for growth. Our analysis suggests that infants who showed a declining trend in LAZ and WAZ scores for the initial months of their life had improvement in these z-scores after 6 months. There were two distinct LAZ trajectories for both boys and girls, while for WAZ, four distinct growth trajectories were observed for both boys and girls, with an initial decline and a later increase in WAZ beyond 6 months. Other studies have observed an increase in WAZ. WAZ growth trajectories that were assessed using latent growth curve models in British and Pakistani children also observed a similar increase after 6 months (31). A similar trend was observed in a study on European children, where children with low birth weight had been shown to catch up after birth (32). This was also similar to growth trajectories observed for BMI z-scores in an Australian birth cohort (15). On the contrary, among Bangladeshi children, there was a declining trend in approximately 95% of the children for LAZ (33). Thus, in general, longitudinal studies have observed an acceleration in growth among children who have started with lower anthropometric measures. This finding contrasts with cross-sectional national surveys, which indicate a decline in growth standards with increasing age and are often used to inform policies for supplementary feeding (1, 16).

We employed latent class mixed models for the latent trajectory identification, which estimates the latent classification based on the cohort being examined and may not be extrapolated to any other population. A similar approach was used to identify growth trajectories in Guatemalan and Australian children (11, 15). Similar to the current study, multiple growth trajectories were identified in the earlier studies. In Guatemalan children, three linear growth trajectories were identified, but all of them had an initial declining slope indicating growth faltering, while in the current study, the predominant trajectory was one without faltering, and this could be because the average LAZ of the Indian children was higher (approximately 1SD at birth) compared to the Guatemalan children (approximately -2SD).

Inadequate child growth has been reported in both urban slums and rural areas of India. An analysis of the National Family Health Survey 4^th^ round (NFHS-4) (2) data showed that a third of the stunted and underweight children belonged to slum areas (34). In NFHS-5, the prevalence of stunting (37% vs. 30%) and wasting (34% vs. 27%) was higher in rural areas compared to urban slums (16). Urban slums and rural areas in India are often associated with poor hygiene and sanitation, which lead to an increased risk of infections and other morbidities. Improper water, sanitation, and hygiene practices have been linked to repeated diarrheal episodes, infections, and environmental enteropathy disease (EED) (35). EED manifests as a subclinical inflammation of the intestines with villous atrophy, potentially leading to poor absorption of nutrients. These episodes of morbidity in children may either reduce the absorption of nutrients or increase their daily nutrient needs.

As discussed earlier, multiple factors influence growth in children. Therefore, exploring these factors can help predict the growth trajectories of infants. In the present analysis, residential location (such as urban and rural settings) and place of birth were found to be significant predictors of LAZ and WAZ trajectories for both boys and girls. The residential area is an important factor for growth as it is linked to hygiene, sanitation, and access to clean drinking water, which could impact episodes of infection and the duration of infection. A study conducted on children (under 24 months) from rural Cambodia (36) showed associations between growth faltering and water, sanitation, and hygiene practices, indicating potential predictors for growth trajectory. Furthermore, in the case of boys, maternal education was found to be a significant predictor for LAZ and WAZ trajectories. Similarly, in other studies, formal education of mothers has been shown to be inversely related to growth faltering (13, 33). Educated mothers are more likely to make informed choices (37) on infant feeding practices that are essential for growth and development. Maternal education was a significant predictor in Indian children, while maternal height was also a significant predictor in Guatemalan children. The Australian children had four trajectories of linear growth.

Although the infants included in these analyses belonged to urban slums and rural areas—environments that may not be conducive to optimal growth—distinct growth trajectories could be identified, which is a notable strength of this study. The predominant trajectories did show a decline in LAZ but showed an increase in WAZ. This contradicts cumulative declining growth curves drawn based on cross-sectional national survey data sets. The data clearly highlight the need for exploring growth patterns with trajectory models to identify subgroups of children with a decline in growth and to implement targeted interventions. The accuracy of the latent trajectories to predict stunting and underweight at 2 years in the same children was found to be poor. Earlier analyses have not explored the ability of latent growth trajectories to predict later nutritional outcomes in children.

### Limitations

Our analysis was limited to the data available from the four selected studies. Therefore, we could not analyze other factors that could impact growth in infants, such as pre-pregnancy weight or BMI, duration of exclusive breastfeeding, complementary feeding practices, duration of infection, parity, maternal age, gestational diabetes, and gestational hypertension. A considerable portion of the original data from the studies were excluded based on the stringent WHO guidelines for cleaning survey data (23) during the data cleaning process (Supplementary Figure 1) because these studies were conducted in populations with a high prevalence of undernutrition.

Another limitation was that the analysis of trajectories with anthropometric outcomes was restricted to a subset of the data, which came from a single study in which data at 2 years were available. Future research with larger and more diverse samples and additional covariates is needed to further investigate the predictive power of early life growth trajectories on later nutritional and health outcomes.

The data used in this study are approximately 20 years old, which limits the interpretation of the results and the implications of our findings for contemporary India. Changes in healthcare, nutrition, and socioeconomic factors over time could impact the relevance of our results. However, this study clearly demonstrates that there is heterogeneity in growth patterns of children in India, and future analysis using more recent data is needed to confirm the applicability of our findings to present-day India.

## Conclusion

In conclusion, our study identified multiple distinct types or classes of longitudinal growth trajectories in Indian children in their first year of life, utilizing a latent class mixed model approach. This finding highlights the heterogeneity of growth patterns, with the predominant class being those who did not experience a decline in LAZ and WAZ. This result contrasts with traditional curve-fit analyses of cross-sectional summary data, where an overall decline in LAZ and WAZ over time is noted in the entire sample of children, disregarding the heterogeneity in growth patterns. These findings demonstrate the growth patterns at a population level, and there is a need for further exploration of growth trajectories using longitudinal cohort data. Future research should focus on validating these trajectories and exploring their predictive value in health outcomes in later life. This research underscores the need for context-sensitive public health interventions with efficient precision public health strategies to improve child nutrition and health outcomes in diverse Indian populations.

## Data Availability

The data analyzed in this study is subject to the following licenses/restrictions: data for the study was accessed through the Healthy Birth, Growth, and Development Knowledge integration (HBGD Ki) platform on 23rd August 2020. Requests to access these datasets should be directed to https://www.kiglobalhealth.org/publications/introduction-to-hbgdki/.
