# Supplementary material for: Growth dynamics of Indian infants using latent trajectory models in pooled survey datasets
Source: Front Public Health. 2025 Jan 7;12:1474222. doi: 10.3389/fpubh.2024.1474222 (PMC11747711; doi:10.3389/fpubh.2024.1474222)
Supplement: Supplementary file 1 [file Table_1.docx]

**Supplementary Note 1: Smoothing of anthropometry data to form monthly data.**

**Supplementary Table 1:** Monthly average z-score of length velocity calculated based on WHO’s LMS parameter.

| Month Interval | Average Z score (SD) | |
| --- | --- | --- |
|  | Boys | Girls |
| 0-2 | -3.48(2.55) | -3.37(2.31) |
| 1-3 | -2.13(3.02) | -2.1(2.75) |
| 2-4 | -1.56(2.33) | -1.58(2.21) |
| 3-5 | -1.32(1.98) | -1.35(1.94) |
| 4-6 | -0.53(1.94) | -0.54(1.98) |
| 5-7 | -0.62(1.7) | -0.64(1.73) |
| 6-8 | -0.62(1.66) | -0.57(1.78) |
| 7-9 | -0.93(1.62) | -0.93(1.71) |
| 8-10 | -1(1.47) | -0.99(1.69) |
| 9-11 | -0.95(1.72) | -1.06(1.8) |
| 10-12 | -0.92(1.79) | -1.04(1.8) |

SD: standard deviation

**Supplementary Table 2:** Monthly average z-score of weight velocity calculated based on WHO’s LMS parameter.

| Month Interval | Average Z score (SD) | |
| --- | --- | --- |
|  | Boys | Girls |
| 0-1 | -1.47(0.99) | -1.88(1.24) |
| 1-2 | -2.15(1.51) | -2.12(1.4) |
| 2-3 | -1(1.88) | -1.14(1.63) |
| 3-4 | -0.99(1.42) | -1.09(1.29) |
| 4-5 | -0.78(1.24) | -0.83(1.18) |
| 5-6 | -0.43(1.48) | -0.49(1.59) |
| 6-7 | -0.41(1.21) | -0.31(1.31) |
| 7-8 | -0.49(1.05) | -0.44(1.08) |
| 8-9 | -0.48(0.96) | -0.34(1.13) |
| 9-10 | -0.37(0.94) | -0.26(1.07) |
| 10-11 | -0.37(0.93) | -0.33(0.96) |
| 11-12 | -0.35(0.88) | -0.3(0.96) |

SD: standard deviation

**Supplementary Table 3**: Selection of optimum number of classes

| **Outcome Variable** | **Class** | **Log-Likelihood** | **BIC** | **% Of children** | | | | |
| --- | --- | --- | --- | --- | --- | --- | --- | --- |
|  |  |  |  | **Class 1** | **Class 2** | **Class 3** | **Class 4** | **Class5** |
| LAZ- Boys  n=1269 | 1 | -12116.97 | 24312.54 |  | 100 |  |  |  |
|  | 2 | -11256.27 | 22648.32 | 83.69 | 16.31 |  |  |  |
|  | 3 | -11093.31 | 22379.57 | 81.25 | 3.23 | 15.52 |  |  |
| LAZ- Girls n=1271 | 1 | -12230.65 | 24539.92 |  |  | 100 |  |  |
|  | 2 | -11339.07 | 22813.93 | 17.07 |  | 82.93 |  |  |
|  | 3 | -11223.29 | 22639.55 | 80.17 | 13.3 | 6.53 |  |  |
|  | 4 | -11147.81 | 22545.78 | 13.3 | 1.02 | 79.62 | 6.06 |  |
| WAZ- Boys  n=1336 | 1 | -12108.15 | 24295.47 | 100 |  |  |  |  |
|  | 2 | -11097.88 | 22332.52 | 19.99 | 80.01 |  |  |  |
|  | 3 | -10881.97 | 21958.28 | 11.53 | 48.35 |  | 40.12 |  |
|  | 4 | -10849.87 | 21951.64 | 46.11 | 36.3 | 11.83 | 5.76 |  |
|  | 5 | -10828.48 | 21966.44 | 5.84 | 11.53 | 12.28 | 24.33 | 46.03 |
| WAZ- Girls n=1354 | 1 | -12863.81 | 25806.93 |  | 100 |  |  |  |
|  | 2 | -11694.84 | 23526.68 | 20.9 | 79.1 |  |  |  |
|  | 3 | -11483.41 | 23161.50 | 14.03 | 56.2 | 29.76 |  |  |
|  | 4 | -11407.57 | 23067.52 | 52.81 | 26.74 | 13.52 | 6.94 |  |
|  | 5 | -11367.59 | 23045.25 | 56.57 | 9.31 | 4.21 | 19.72 | 10.19 |

BIC: Bayesian Information Criterion; LAZ: Length for Age Z score; WAZ: Weight for Age Z score

**Supplementary Table 4. Distribution of Latent class membership by intervention group in two studies**

| SAS Food Supply |  |  |  | SAS Comp feed |  |  | Zn-SGA |  |  |
| --- | --- | --- | --- | --- | --- | --- | --- | --- | --- |
| HAZ Female |  | n(% in Class) | n(% in Class) |  | n(% in Class) | n(% in Class) |  | n(% in Class) | n(% in Class) |
|  | Latent Class | Control | Intervention | Latent Class | Control | Intervention | Latent Class | Control | Intervention |
|  | Class 1 | 102(91.89%) | 98(92.45%) | Class 1 | 189(98.95%) | 195(97.5%) | Class 1 | 53(39.55%) | 40(25.32%) |
|  | Class 2 | 2(1.8%) | 5(4.72%) | Class 2 | 2(1.05%) | 5(2.5%) | Class 2 | 45(33.58%) | 84(53.16%) |
|  | Class 3 | 7(6.31%) | 3(2.83%) | Class 3* |  |  | Class 3 | 36(26.86%) | 34(21.52%) |
| P value | 0.24 |  |  | 0.483 |  |  | 0.003 |  |  |
| HAZ Male |  |  |  |  |  |  |  |  |  |
|  | Class 1 | 84(89.36%) | 94(96.91%) | Class 1 | 211(89.36%) | 20(96.91%) | Class 1 | 75(68.18%) | 75(65.57%) |
|  | Class 2 | 10(10.64%) | 3(3.1%) | Class 2 | 2(10.64%) | 2(3.09%) | Class 2 | 35(31.81%) | 36(32.43%) |
| P value | 0.075 |  |  | 1 |  |  | 1 |  |  |
| WAZ Male | Class 1 | 39(41.05%) | 32(32.99%) | Class 1 | 136(63.55%) | 133(63.33%) | Class 1 | 10(6.89%) | 10(7.69%) |
|  | Class 2 | 51(53.68%) | 60(61.86%) | Class 2 | 72(33.645%) | 75(35.71%) | Class 2 | 36(24.83%) | 32(24.61%) |
|  | Class 3 | 3(3.16%) | 2(2.06%) | Class 3 | 6(2.81%) | 2(0.95%) | Class 3 | 72(49.65%) | 61(46.92%) |
|  | Class 4 | 2(2.10%) | 3(3.1%) | Class 4* |  |  | Class 4 | 27(18.62%) | 27(20.77%) |
| P value | 0.615 |  |  | 0.357 |  |  | 0.955 |  |  |
|  | Class 1 | 72(65.45%) | 64(59.26%) | Class 1 | 116(60.42%) | 134(65.68%) | Class 1 | 43(25.44%) | 56(29.63%) |
| WAZ Female | Class 2 | 30(27.27%) | 31(28.7%) | Class 2 | 70(36.46%) | 61(29.9%) | Class 2 | 23(13.61%) | 12(6.35%) |
|  | Class 3 | 8(7.27%) | 3(12.04%) | Class 3 | 6(3.16%) | 8(3.92%) | Class 3 | 59(34.91%) | 73(38.63%) |
|  | Class 4* |  |  | Class 4 | 0(0%) | 1(0.49%) | Class 4 | 44(26.03%) | 48(25.4%) |
| P value | 0.436 |  |  | 0.417 |  |  | 0.125 |  |  |

* there are no children from the study belonging to the corresponding trajectory class.

**Supplementary Table 5:** Class membership model for LAZ

|  |  | Boys | Girls | |
| --- | --- | --- | --- | --- |
|  |  | Class 1 Est (95% CI) | Class 1 Est (95% CI) | Class 2 Est (95% CI) |
| Intercept |  | -3.08  (-4.49, -1.67) | -12.44  (-233.85, 208.97) | 14.1  (-350.68, 378.89) |
| Residential area | Rural | *Ref* | | |
|  | Urban slum | 3.34 (2.61, 4.07) | 11 (-214.89, 236.88) | -3.2 (-4.18, -2.21) |
| Place of birth | Institutional | *Ref* | | |
|  | Home | 0.78 (0.36, 1.21) | 0.52 (-0.23, 1.27) | -0.51 (-0.99, -0.04) |
| Mother Education Level | College | *Ref* | | |
|  | Schooling | -1.61  (-2.95, -0.26) | 0.43 (-7.96, 8.83) | -9.65  (-374.27, 354.97) |
|  | Illiterate | -1.37  (-2.72, -0.02) | 0.1 (-8.24, 8.44) | -9.87  (-374.51, 354.77) |

Class of reference is the last class.

**Supplementary Table 6:** Class membership model for WAZ

|  |  | Boys | | | Girls | | |
| --- | --- | --- | --- | --- | --- | --- | --- |
|  |  | Class 1 Est (95% CI) | Class 2 Est (95% CI) | Class 3 Est (95% CI) | Class 1 Est (95% CI) | Class 2 Est  (95% CI) | Class 3 Est (95% CI) |
| Intercept |  | 2.18  (0.26, 4.1) | -5.42  (-51.44, 40.61) | -0.08  (-2.39, 2.22) | 1.47  (-89.06, 92) | 12.66  (-95.69, 121.01) | 0.43  (-22.32, 23.18) |
| Residential area | Rural | *Ref* | | | | | |
|  | Urban slum | -3.89  (-4.81, -2.98) | -2.99  (-3.91, -2.06) | 2.33  (-1.5, 6.16) | -4.01  (-6.53, -1.5) | -3.69  (-6.05, -1.34) | -1.42  (-4.16, 1.32) |
| Place of birth | Institutional | *Ref* | | | | | |
|  | Home | -1.19  (-1.71, -0.67) | -0.93  (-1.46, -0.4) | -0.25  (-1.14, 0.65) | -0.78  (-1.49, -0.08) | -1.46  (-2.11, -0.81) | -0.19  (-1.02, 0.63) |
| Mother Education Level | College | *Ref* | | | | | |
|  | Schooling | 3.02  (0.84, 5.2) | 9.29  (-36.74, 55.32) | -2.58  (-5.61, 0.45) | 3.7  (-86.95, 94.34) | -6.89  (-115.42, 101.64) | 1.69  (-21, 24.37) |
|  | Illiterate | 2.23 (0.04, 4.43) | 9.43  (-36.61, 55.46) | -2.81 (-5.83, 0.22) | 3.53  (-87.13, 94.2) | -6.65  (-115.19, 101.89) | 1.86  (-20.81, 24.52) |

Class of reference is the last class

**Supplementary Table 7:** Distribution of early life characteristics of children over latent class trajectories of LAZ

| **Characteristics** |  | **LAZ-Boys** | | **LAZ-Girls** | | |
| --- | --- | --- | --- | --- | --- | --- |
|  |  | **Class 1**  **N (%)** | **Class 2**  **N (%)** | **Class 1**  **N (%)** | **Class 2**  **N (%)** | **Class3**  **N (%)** |
| Residential area | Rural | 653(61.49) | 10(4.83) | 563(55.25) | 9(5.33) | 0(0) |
|  | Urban slum | 409(38.51) | 197(95.17) | 456(44.75) | 160(94.67) | 83(100) |
| Place of birth | Institutional | 306(28.81) | 58(28.02) | 265(26.01) | 56(33.14) | 19(22.89) |
|  | Home | 756(71.19) | 149(71.98) | 754(73.99) | 113(66.86) | 64(77.11) |
| Mother's Educational Level | College | 14(1.32) | 5(2.42) | 11(1.08) | 0(0) | 0(0) |
|  | Schooling | 495(46.61) | 64(30.92) | 456(44.75) | 54(31.95) | 31(37.35) |
|  | Illiterate | 553(52.07) | 138(66.67) | 552(54.17) | 115(68.05) | 52(62.65) |

LAZ: Length for Age Z score

**Supplementary Table 8:** Distribution of early life characteristics of children over latent class trajectories of WAZ

| **Characteristics** | | **WAZ-Boys** | | | | **WAZ-Girls** | | | |
| --- | --- | --- | --- | --- | --- | --- | --- | --- | --- |
|  |  | **Class 1**  **N (%)** | **Class 2 N (%)** | **Class3 N (%)** | **Class4N (%)** | **Class 1 N (%)** | **Class 2 N (%)** | **Class3 N (%)** | **Class4 N (%)** |
| Residential area | Rural | 445  (72.3) | 209  (43.1) | 10  (6.33) | 1  (1.3) | 324  (45.31) | 234  (64.64) | 17  (9.29) | 2  (2.13) |
|  | Urban slum | 171  (27.7) | 276  (56.9) | 148  (93.7) | 76  (98.7) | 391  (54.69) | 128  (35.36) | 166  (90.71) | 92  (97.87) |
| Place of birth | Institutional | 181  (29.4) | 139  (28.6) | 39  (24.7) | 25  (32.5) | 240  (33.57) | 64  (17.68) | 38  (20.77) | 20  (21.28) |
|  | Home | 435  (70.6) | 346  (71.3) | 119  (75.3) | 52  (67.5) | 475  (66.43) | 298  (82.32) | 145  (79.23) | 74  (78.72) |
| Mother’s Educational Level | College | 10  (1.62) | 0  (0) | 3  (1.9) | 9  (11.7) | 12  (1.68) | 0(0) | 0  (0) | 0  (0) |
|  | Schooling | 364  (59.1) | 151  (31.2) | 47  (29.7) | 26  (33.7) | 288  (40.28) | 187  (51.66) | 64  (34.97) | 33  (35.11) |
|  | Illiterate | 242  (39.3) | 334  (68.9) | 108  (68.4) | 42  (54.5) | 415  (58.04) | 175  (48.34) | 119  (65.03) | 61  (64.89) |

WAZ-Weight for Age Z score

**Supplementary Table 9:** Associations of LAZ growth latent classes and stunting outcome variables

| Variable | Category | Stunting boys | Stunting girls | Underweight boys | Underweight girls |
| --- | --- | --- | --- | --- | --- |
|  |  | OR (95% CI) | OR (95% CI) | OR (95% CI) | OR (95% CI) |
| Intercept |  | 28.5 | 5.93 | 2.23 | 0.82 |
|  |  | (3.37 ,241.39) | (1.36 ,25.79) | (0.63 ,7.8) | (0.25,2.65) |
| Class membership probability | latent class1 |  | 0.79 |  |  |
|  |  |  | (0.26 ,2.36) |  |  |
|  | latent class2 | 1.67 |  | 12.43 | 7.24 |
|  |  | (0.46 ,6.07) |  | (4.85 ,31.84) | (1.96,26.93) |
|  | latent class3 |  |  | 6.36 | 5.26 |
|  |  |  |  | (1.25 ,32.36) | (0.85,32.55) |
|  | latent class 4 |  |  | 1.06 |  |
|  |  |  |  | (0.22 ,5.09) |  |
| Birth weight | Normal | Ref | | | |
|  | Low birth weight | 0.16 | 0.68 | 0.09 | 0.1 |
|  |  | (0.02 ,1.22) | (0.23 ,2.05) | (0.03 ,0.29) | (0.03,0.36) |
| Mother’s educational Level | Illiterate | Ref | | | |
|  | Schooling or college | 0.43 | 0.43 | 0.93 | 1.19 |
|  |  | (0.18 ,1.06) | (0.2,0.92) | (0.43 ,2) | (0.59,2.4) |

OR: Odds ratio

**Supplementary Fgure 1**: Sample size selection flow chart for the analysis

Final Sample Size for LAZ trajectory model

(N=16822 NC=2540)

Final Sample Size for WAZ trajectory model

(N=24268 NC=2690)

Combined

(N=40584 NC=4323)

CMC V BC 2002

(N=17082 NC=373)

SAS comp feed

(N=9541 NC=1535)

SAS food supply

(N=2249 NC=418)

Zn-SGA

(N= 11712 NC=1997)

Less than 12 months

(N=26661 NC=4323)

Removal of outliers for WAZ

(N=2393 NC=1633)

Removal of outliers for LAZ

(N=9839 NC=1783)

N- number of observations and NC- number of unique child.

**Supplementary Figure 2** Study wise distribution of LAZ and WAZ


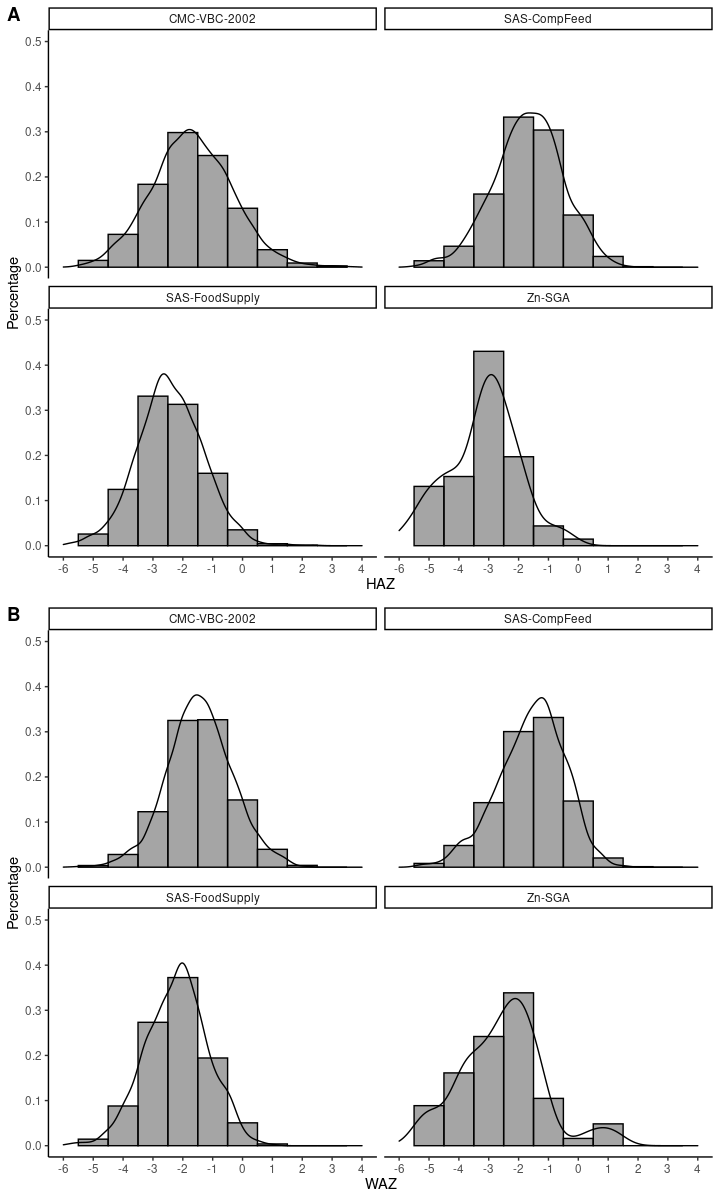


Distribution of length-for-age z-scores (LAZ) and weight-for-age z-scores (WAZ) among infants across different studies. Panel A displays the distribution of LAZ, while Panel B illustrates the distribution of WAZ

**Technical description of LCMM (Latent class mixed models)**

Latent class mixed models (LCMM) are a statistical approach used to analyze longitudinal data, particularly when there are unobserved (latent) groups or classes within the data. LCMM combines elements of latent class analysis (LCA) and mixed-effects models to identify these latent classes and model their development over time.

LCMM assumes that there are unobserved (latent) classes within the data, each characterized by a distinct pattern of growth trajectories. The number of latent classes is typically unknown and is determined based on statistical criteria or theoretical considerations. Within each latent class, the growth trajectories of individuals are modeled using mixed-effects models. These models account for the correlation between repeated measurements within individuals and allow for the estimation of both fixed effects (population-level parameters) and random effects (individual-level deviations from the population-level parameters). Once the model is fitted, the latent classes and their associated growth trajectories can be interpreted to understand the different patterns of growth within the population. Additionally, the model can be used to predict individual trajectories and assess the impact of covariates on these trajectories.

In our case , this approach facilitates the identification of latent trajectories which are unobservable by usual longitudinal analysis which utilizes the average anthropometric measure across time points. The method considered the interdependency in growth measurements between time points by the mixed model approach where the covariance between the measurements is considered which is an improvement over the simpler clustering approach.

Overall, LCMM is a flexible and powerful approach for analyzing longitudinal data with complex patterns of growth, allowing researchers to uncover hidden structures within the data and gain insights into the underlying processes driving development over time.
